# STING Is Required in Conventional Dendritic Cells for DNA Vaccine Induction of Type I T Helper Cell- Dependent Antibody Responses

**DOI:** 10.3389/fimmu.2022.861710

**Published:** 2022-04-22

**Authors:** Justin Theophilus Ulrich-Lewis, Kevin E. Draves, Kelsey Roe, Megan A. O’Connor, Edward A. Clark, Deborah Heydenburg Fuller

**Affiliations:** ^1^ Department of Microbiology, University of Washington, Seattle, WA, United States; ^2^ Department of Immunology, University of Washington, Seattle, WA, United States; ^3^ Seattle Children's Hospital Center for Immunity and Immunotherapies Children’s Hospital, Seattle, WA, United States

**Keywords:** DNA vaccine, *STING*, dendritic cells, type I interferon, *cGAS*

## Abstract

DNA vaccines elicit antibody, T helper cell responses and CD8^+^ T cell responses. Currently, little is known about the mechanism that DNA vaccines employ to induce adaptive immune responses. Prior studies have demonstrated that *stimulator of interferon genes* (*STING*) and conventional dendritic cells (cDCs) play critical roles in DNA vaccine induced antibody and T cell responses. *STING* activation by double stranded (dsDNA) sensing proteins initiate the production of type I interferon (IFN),but the DC-intrinsic effect of *STING* signaling is still unclear. Here, we investigated the role of *STING* within cDCs on DNA vaccine induction of antibody and T cell responses. *STING* knockout (*STING^-/-^*) and conditional knockout mice that lack *STING* in cDCs (*cDC STING cKO*), were immunized intramuscularly with a DNA vaccine that expressed influenza A nucleoprotein (pNP). Both *STING^-/-^* and *cDC STING cKO* mice had significantly lower type I T helper (Th1) type antibody (anti-NP IgG_2C_) responses and lower frequencies of Th1 associated T cells (NP-specific IFN-γ^+^CD4^+^ T cells) post-immunization than wild type (WT) and *cDC STING littermate control* mice. In contrast, all mice had similar Th2-type NP-specific (IgG_1_) antibody titers. *STING^-/-^* mice developed significantly lower polyfunctional CD8^+^ T cells than WT, *cDC STING cKO* and *cDC STING littermate control* mice. These findings suggest that *STING* within cDCs mediates DNA vaccine induction of type I T helper responses including IFN-γ^+^CD4^+^ T cells, and Th1-type IgG_2C_ antibody responses. The induction of CD8^+^ effector cell responses also require *STING*, but not within cDCs. These findings are the first to show that *STING* is required within cDCs to mediate DNA vaccine induced Th1 immune responses and provide new insight into the mechanism whereby DNA vaccines induce Th1 responses.

## Introduction

DNA vaccines induce robust antibody (Ab) and T cell responses in small animals, but their immunogenicity in humans has generally been much lower and often below the threshold needed for protection (1–5). To improve DNA vaccine potency, it is essential to understand the mechanisms that govern DNA vaccine immunogenicity. However, to date the innate immune pathways that are triggered by DNA vaccines and the influence these pathways have on DNA vaccine induced immune responses have not been fully elucidated. Previous studies hypothesized that unmethylated CpG motifs within the DNA plasmid can activate *toll like receptor 9* (*TLR9*), an endosomal dsDNA receptor, to program vaccine induced immune responses. However, these studies showed that *TLR9*, and its downstream adaptor protein, *myeloid differentiation primary response gene 88* (*MyD88*) were not required to mediate DNA vaccine immunogenicity (6).

Double stranded DNA (dsDNA) sensing pathways have been hypothesized to be key mediators of DNA vaccine immunogenicity. Specifically, *cyclic GMP-AMP synthase* (*cGAS*), binds to dsDNA within the cytoplasm and induces the production of type I interferon (IFN) responses *via stimulator of interferon genes* (*STING*). However, studies to determine if *cGAS* is a major regulator of DNA vaccine immunogenicity, showed that it is dispensable for DNA vaccine induced immune responses, whereas *STING* (*stimulator of interferon genes*) and other proteins downstream of *STING* including *TANK-binding kinase 1* (*TBK1*) and *interferon regulatory factor 7* (*IRF7*) were required (7–9).

Here, we explored the mechanism by which *STING* governs downstream DNA vaccine immunogenicity including the induction Th1 and Th2-biased Ab and polyfunctional CD8^+^ T cell responses. Previous studies showed that conventional dendritic cells (cDCs) play a central role in coordinating DNA vaccine induction of Ab and T cell responses (10–14). Our studies confirm these finding and further show that *STING* is required within cDCs to mediate DNA vaccine induction of Th1 CD4^+^ T cells secreting IFN-γ and Th1-associated IgG_2C_ Ab responses, but not Th2 type IgG_1_ Ab responses. Interestingly, these studies also show that while *STING* is required for the induction of polyfunctional CD8^+^ T cell responses, it is not required within cDCs. These results provide new insight into the mechanisms whereby *STING* and cDCs mediate DNA vaccine induction of immune responses. These findings have implications for the development of new adjuvants targeting *STING* to enhance DNA vaccine immunogenicity.

## Materials and Methods

### Mice

C57BL/6J (WT), *STING ^-/-^*, and *cGAS ^-/-^* mice were bred and maintained in-house at the University of Washington. *Sting^fl/fl^* mice were a generous gift from Dr. Mohamed Oukka (University of Washington, Seattle, Washington). *STING^fl/fl^* mice were made by a commercial service (Biocytogen, Wakefield MA) using a Cas9/sgRNA plasmid construct. In brief, a targeting vector was designed with a Neo cassette, flanked by Frt sites, and *STING* Exon 6 flanked by LoxP sites, was introduced into a B6-derived embryonic stem cells (ES). Targeted ES cells were introduced into host embryos, and cell embryos were surgically transferred into pseudo-pregnant (surrogate) mothers resulting in F0 heterozygous floxed mice on the B6 background. Chimeric mice were crossed to B6J (JAX #000664) for 6 generations. *STING^fl/fl^* mice were bred to *zbtb46* ^Cre^ mice (JAX #028538) to generate *zbtb46^Cre^* x *STING^f/fl/^* mice (*cDC STING cKO*). The *zbtb46^Cre^* x *STING^f/fl^* genotyping was carried out using tail snips followed by PCR (**Supplementary Table 1**). *Zbtb46* is expressed by cDCs, but not plasmacytoid DCs (15), and as such *cDC STING cKO* mice should have STING selectively absent in cDCs. We verified that cDCs in *cDC STING cKO* mice were missing *STING via* qPCR for the detection of *STING* (**Supplementary Figure 1**). Three to five *STING^fl/fl^, zbtb46^Cre^* and *cDC STING cKO* mice were sacrificed, spleens were collected, pooled, and made into single cell suspensions by crushing spleens through a 0.70μm filter followed by a 20ml wash with full RPMI media (RMPI 1640 supplemented with 10% FBS, non-essential amino acids, sodium pyruvate, pen/strep and β-mercaptoethanol). Red blood cells were lysed using an RBC lysis buffer (Thermofisher cat. no. 00-4300-54). Splenocytes were stained with Live/Dead fixable aqua dead (ThermoFisher Scientic cat. no. L34957), PerCP-conjugated anti-mouse CD11c (Biolegend cat. no. 117326), BV605-conjugated anti-mouse CD19 (Biolegend cat. no. 115540), BV605-conjugated anti-mouse CD3 (Biolegend cat. no. 100237), APC-conjugated anti-mouse Ly-6G (Biolegend cat. no. 127614), PE-conjugated anti-mouse NK1.1 (Biolegend cat. no. 108708), APC-Cy7-conjugated anti-mouse CD11c, and Pacific blue-conjugated anti-mouse MHC-II (Biolegend cat. no. 107620). A BD Biosciences FACS ARIA III cell sorter was used to isolate cDC (CD11c^+^MHCII^hi^), and B and T cells (CD19^+^CD3^+^). RNA was isolated (Qiagen RNeasy kit cat. no. 74004) and then converted to cDNA (ThermoFisher cat. no. 4368814). *STING* expression in each cell type was measured *via* qPCR using primer F: GGGAGCCGAAGACTGTACAT; primer R: CGCTGTTGGAAAAACCCGA. All mice were maintained by the department of animal welfare (OAW) according to institutional Animal Care and Use Committee (IACUC)- approved protocols.

### DNA Vaccination

A DNA vaccine that encodes a codon-optimized full-length nucleoprotein from A/Puerto Rico/8/1934 (pNP) was used to immunize mice. Construction of the plasmid, pNP, employed for these studies is previously described (16). Briefly, the influenza NP gene is under control of the human cytomegalovirus (CMV) immediate early promoter. This plasmid also includes the following additional elements to optimize antigen expression: the hepatitis B virus (HBV) pre-S2 5’ untranslated region (UTR), rabbit beta globin poly A, rat insulin intron A, the HBV env enhancer and the CMV exon 1 and 2. Mice were injected with 10 μg of plasmid nucleoprotein (pNP) in 50μl PBS split between both tibialis anterior, and then were electroporated using a BTX agilepulse waveform electroporation system (cat. no. 47-0400N). BTX’s agilepulse voltage and pulse length settings used for intramuscular (IM) vaccinations were as follows: Group 1; 450 V pulse amplitude, 0.05ms pulse width, 300ms pulse interval, 500ms group intervals, 2 pulses, and Group 2; 110V pulse amplitude, 10ms pulse width, 300ms pulse interval, 500ms group interval, 8 pulses. Gene gun (GG) DNA vaccination was also used to vaccinate mice with pNP. Mice were vaccinated with 1μg pNP using helium at 400psi (**Supplementary Figure 2**).

### ELISA

Sera were collected at 14-, 21- and 28-days post-vaccination to measure NP specific IgG, IgG_1_, and IgG_2C_ antibody titers, and sera samples were stored at -80°C until the time of assay. Microtiter plates were coated at 1μg/ml of recombinant NP (Sino Biological inc, cat. no 11675-V08B) overnight at 4°C. Anti-NP IgG, IgG1 and IgG_2C_ concentrations (μg/ml) in sera were calculated using purified mouse IgG (Southern Biotech cat. no. 0107-01), IgG_1_ (Southern Biotech cat. no. 0102-01), or IgG_2C_ (Southern Biotech cat. no. 1078-01) to produce a standard curve. Purified mouse antibodies were detected using 1μg/ml of goat anti-mouse IgG-HRP (Southern Biotech cat. no. 1030-05), IgG_1_-HRP (Southern Biotech cat. no. 1070-05) or IgG_2C_–HRP (Sothern Biotech cat. no. 1078-05), respectively. O-phenylenediamine dihydrochloride (OPD) substrate tablets (ThermoFisher cat. no. 34006) were used to observe HRP induced color changes. The HRP substrate reaction was terminated using 2N sulfuric acid after 2 minutes. Absorbance was measured at 450nm and concentrations of antibody were calculated using each plate’s internal standard curve. Sera dilutions were optimized for each experiment and timepoint to ensure absorbance reading fell within each plates internal standard curve. Data are representative of at least three independently preformed studies (n=3-9 mice/genotype/study).

### Analysis of Antigen-Specific T Cell Responses Tetramer Staining and Flow Cytometry

Antigen (Ag)-specific CD8^+^ (**Supplementary Figure 3**) and CD4^+^ T cells (**Supplementary Figure 5**) were measured by flow cytometry using PE-and/or APC-conjugated-NP_366-374_ (H-2k(b)) and PE-conjugated- NP_311-325_ (I-A(b)) tetramers, respectively. Tetramers were obtained from the NIH tetramer core facility (Atlanta, GA USA). Mice were sacrificed, spleens collected and single cell suspensions were generated for each mouse as outlined above. A total of 1x10^6^ splenocytes were stained and analyzed *via* flow cytometry (BD bioscience LSRII). To measure the frequencies of Ag-specific CD4^+^ T cells, splenocytes were stained with the following antibodies: Live/Dead fixable aqua dead (ThermoFisher Scientic cat.no. L34957), BUV395-conjugated anti-mouse CD4 (BD cat. no. 565975), PerCP5.5-conjugated anti-mouse CD3 (Biolegend cat. no. 1002180), BV711-conjugated anti-mouse CD8 (Biolegend cat. no. 100748), PE-Cy7-conjugated anti-mouse CD44 (BD cat. no. 560569) and PE-conjugated- NP_311-325_. To measure the frequencies of Ag-specific CD8^+^ cells, splenocytes were stained as follows: Live/Dead fixable aqua dead (ThermoFisher Scientic cat. no. L34957), PerCP5.5-conjugated anti-mouse CD3 (Biolegend cat. no. 1002180), BUV395-conjugated anti-mouse CD4 (BD cat. no. 565975), BV711-conjugated anti-mouse CD8 (Biolegend cat. no. 100748) and PE-Cy7-conjugated anti-mouse CD44 (BD cat. no. 560569).

### Analysis of Polyfunctional CD8^+^ T Cell Responses

Single cell suspensions from spleens were collected 21 days post-vaccination as outlined above then 1x10^6^ cells were plated in duplicate wells in a 96 well plate and rested for 24 hours (hrs.) in full RPMI media (details above) at 37°C. To evaluate CD8^+^ T cell polyfunctionality cells were either stimulated with 1μg/ml NP_366-374,_ or media only (unstimulated). After one hour, 1x brefeldin A (Biolegend cat. no. 420601) and FITC-conjugated CD107a/GranzymeB (Biolegned cat. no. 121606) were added to the wells and incubated overnight (~16hrs). The following day, cells were stained for IL-2 (Biolegend cat. no. 503808), IFN-γ (Biolegend cat. no. 505818), FITC-conjugated anti-human/mouse Granzyme B (Biolegend cat. no. 515403) and TNF-α (Biolegend cat.no 506308) using BD’s fixation/permeabilization solution kit (cat.no. 554714) and the manufacture’s protocol. T cells were first gated using BV605-conjugated anti-mouse CD3 (Biolegend cat. no. 100237). CD8^+^ T cells were gated using BV711-conjugated anti-mouse CD8 (Biolegend cat. no. 100748). The frequencies of CD8^+^ T cells expressing IL-2, TNF-α, IFN-γ and/or cytolytic markers CD107a/Granzyme B were then measured by flow cytometry (BD Biosciences LSRII) and Boolean gating (**Supplementary Figure 4**). The frequencies of mouse CD8^+^ T cell in media were subtracted from their frequencies following NP peptide stimulation. These data were then used to calculate a polyfunctional index as described (17). CD8^+^ T cell polyfunctionality is defined as the frequency of CD8^+^ T cells expressing any three or more of the cytokines IFN-γ, IL-2, TNF-α and/or co-expressing the cytolytic marker CD107a/GranzymeB after stimulation with the immunodominant NP peptide, NP_366-374_, specific for C57BL/6 mice.

### Analysis of Th1 and Th2 Associated Cytokines Produced by Peptide Stimulated Splenocytes

To measure Th1 and Th2 cytokine expression, 1x10^6^ cells were plated in duplicate, rested for 24hrs in full media at 37°C and then one well was stimulated overnight (~16hrs) with 1μg/ml NP_311-325_. Supernatants were collected and analyzed for IFN-γ, TNF-α, IL-2, IL-4, IL-6, IL-10 and IL-17a *via* flow cytometry (BD Bioscience LSRII) using BD’s mouse Th1/Th2/Th17 Cytometric Bead Array kit (cat. no. 560485) using the manufacture’s protocol.

### Analysis of IFN-γ Expression in Peptide Stimulated CD4^+^ T Cells

To measure IFN-γ expressing CD4^+^ T cells mouse splenocytes were isolated as outlined above. A total of 1x10^6^ cells were pated in duplicate in a 96 well plate and rested for 24hrs. The cells were then stimulated with 1μg/ml NP_311-325_, the immunodominant NP peptide for C57BL/6 CD4^+^ T cells or remained in media only. After one hour, 1x brefeldin A (Biolegend cat. no. 420601) was added to wells and the cells were incubated overnight (~16hrs). The following day splenocytes were stained with PerCP5.5-conjugated anti-mouse CD3 (Biolegend cat. no. 1002180), BUV395-conjugated anti-mouse CD4 (BD cat. no. 565975) and IFN-γ (Biolegend cat. no. 505818) using BD’s fixation/permeabilization solution kit (cat. no. 557414) and the manufacture’s protocol. The frequencies of CD4^+^ T cells expressing IFN-γ was measured by flow cytometry (DB Bioscience LRSII).

### B Cell ELISPOT

Mouse splenocytes were used to enumerate NP-specific antibody secreting cells 21 days post-vaccination. ELISPOT plates (Millipore Sigma cat. no. S2EM004M99) were coated with 10μg/ml recombinant NP (Sino Biological inc, cat. no 11675-V08B) in PBS and then incubated overnight at 4°C. Mouse splenocytes were processed as outlined above and then 3x10^6^ splenocytes in 100μl of media were plated in NP-coated plates in duplicate and then incubated overnight (~16hrs) at 37°C. The following day, the cells were discarded and the wells were washed 5 times with PBS. Following the final wash, 100μl of anti-mouse IgG-HRP, IgG_1_-HRP, or IgG_2C_–HRP diluted at 1:2000 in PBS were added to each well. Spots were developed using BD ELISPOT AEC substrate set (BD cat. no. 551951) and then visualized using a CTL imager (Cellular Technologies) and were enumerated using an ImageJ cell counter.

### Cytokine Analysis

Sera were collected from mice prior to vaccination (baseline), and at 6hrs and 24hrs post-vaccination. Sera from WT, *zbtb46^Cre^ x STING^fl/fl^* (*cDC STING cKO*) and *cDC STING LitC* mice were diluted 1:5 while sera from *STING^-/-^* mice were diluted 1:2. Levels of IFN-γ, TNF, IL-2, IL-4, IL-6, IL-10 and IL-17a in diluted sera were then measured by flow cytometry (BD Bioscience LSRII) using BS’s mouse Th1/Th2/Th17 Cytometric Bead Array kit (cat. no. 560485) following the manufacture’s protocol. IFN beta (IFN-β) was measured in sera diluted 1:2 prior to vaccination and 6hrs post-vaccination using mouse IFN-β ELISA kit (PBL Assays cat. no. 42410-1).

### Statistical Analysis

All data are represented as the mean of individual mice ± SD. Statistical analyses were performed using one-way ANOVA followed by a turkey post-test and Student’s t test using Graphpad Prism 7.

## Results

### *STING*, But Not *cGAS*, Is Required to Mediate DNA Vaccine Induction of Th1 Associated IgG_2C_ Ab Responses

**Figure 1:**
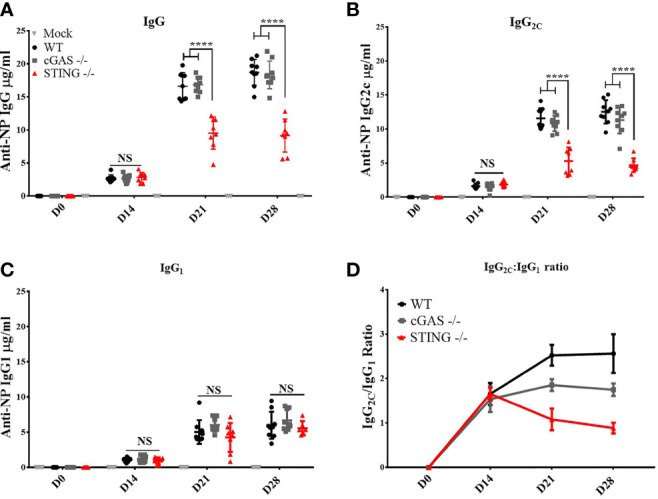
*STING* is required for DNA vaccine induction of antigen-specific Th1 associated IgG_2C_ antibody responses. Wild type (WT), *cGAS^-/-^* and *STING* ^-/-^ mice were IM/EP vaccinated with a DNA vaccine (pNP) expressing influenza nucleoprotein (pNP). Sera were collected prior to vaccination (D0), and at 14 (D14), 21 (D21), and 28 days post-vaccination (D28). The concentration of anti-NP **(A)** IgG, **(B)** IgG_2C_ and **(C)** IgG_1_ antibody responses was measured by ELISA. Each ELISA plate used goat anti-mouse IgG and known concentrations of mouse IgG, IgG_2C_, or IgG_1_ to create a standard curve to back calculate antibody titers (μg/ml). **(D)** The Th1:Th2 ratio was calculated as IgG_2C_:IgG_1_, a higher ratio is indicative of a Th1 response. Three independent experiments consisting of 3-9 mice were performed consisting of 3-9; representative data are the average ±SD 3-9 mice/genotype. At each timepoint a one-way ANOVA was used. ****p < 0.0001 and NS, not significant.

To determine if *cGAS* and/or *STING* play a role in the ability of DNA vaccines to induce antibody (Ab) responses, WT, *cGAS^-/-^* and *STING^-/-^* mice were vaccinated by intramuscular delivery and electroporation (IM/EP) with 10μg of a codon optimized DNA vaccine expressing influenza A nucleoprotein (pNP). Sera were collected 14-, 21- and 28-days post-vaccination to analyze induction of NP-specific IgG Ab responses. *STING^-/-^* mice generated significantly lower anti-NP IgG Ab concentrations at 21- and 28-days post-vaccination compared to WT and *cGAS^-/-^* mice (**Figure 1A**), a result that confirms previous studies that *STING*, but not *cGAS*, plays a role in DNA vaccine immunogenicity (9). The lower IgG titers in *STING^-/-^* mice led us to investigate if both Th1 (IgG_2C_) and Th2 (IgG_1_) Ab responses were impacted by *STING* or *cGAS*. Analysis of IgG_2C_ and IgG_1_ titers, and IgG_2C_/IgG_1_ ratios in vaccinated WT, *cGAS^-/-^* and *STING^-/-^* mice showed that *STING^-/-^* mice developed significantly lower Th1 associated anti-NP IgG_2C_ (**Figure 1B**) than WT and *cGAS^-/-^* mice, but comparable levels of Th2 associated NP-specific IgG1 Ab titers (**Figure 1C**). A similar outcome was observed in *cGAS^-/-^*, *STING^-/-^* and WT mice immunized with the same DNA vaccine by gene gun indicating that these results are not dependent on the route of DNA vaccine delivery (**Supplementary Figure 2**). Taken together, these data are consistent with previous findings showing that *STING* is required to induce Ab responses, but further shows that *STING* is necessary to generate Th1 IgG_2C_ antibody, but not Th2 IgG_1_ responses.

### STING Is Required for DNA Vaccine Induction of Polyfunctional CD8^+^ T Cell Responses

**Figure 2:**
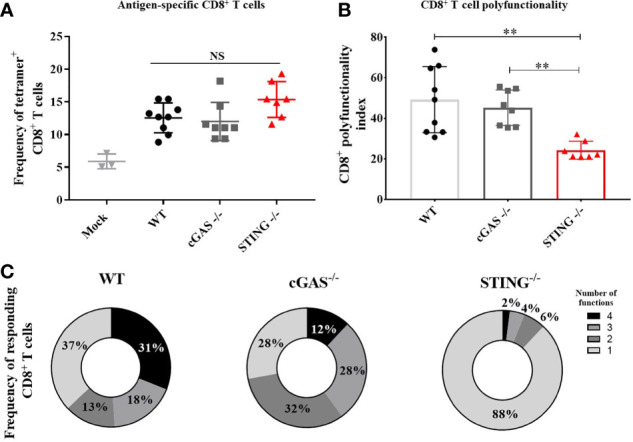
*STING* is required for DNA vaccine induction of antigen-specific CD8^+^ T cell polyfunctionality. WT, *cGAS^-/-^* and *STING^-/-^* mice were IM/EP vaccinated with pNP. Mice were sacrificed 21-days post-vaccination and splenocytes were isolated and made into single cell suspensions. **(A)** The frequency of tetramer positive cells within CD8^+^ T cells were measured for each genotype using a MHC-I tetramer that presents the NP immunodominant peptide (NP_366-374_) for C57bl/6. CD8^+^ T cells and cells were gated as outlined in **Supplementary Figure 3B**. **(B)** Polyfunctional scores were determined by stimulating splenocytes overnight with 1μg/ml of NP_366-375_ followed by ICS staining for IL-2, TNF-α, IFN-γ and CD107a/GranzymeB and analyzed by flow cytometry as shown in **Supplementary Figure 4**. Polyfunctionality scores were calculated as described in the methods and Larsen et al (17). **(C)** Pie charts show the relative average proportion of CD8^+^ T cells producing 1-4 immune factors (IL-2, TNF-α, IFN-γ and/or CD107a/GranzymeB) after NP_366-374_ stimulation. Three independent experiments consisting of 3-9 mice were performed consisting of 3-9 mice; representative data are the average± SD of 3-9 mice/genotype. Groups were compared using a one-way ANOVA, **p<0.01 and NS, not significant.

A hallmark of DNA vaccines is their ability to induce Th1 responses that mediate the induction of CD8^+^ T cell responses including cytotoxic T lymphocytes (2–5, 18). To determine if *STING* or *cGAS* play a role in DNA vaccine induction of CD8^+^ T cell responses, WT, *cGAS^-/-^* and *STING^-/-^* mice were vaccinated with pNP by IM/EP vaccine delivery. Mice were sacrificed 21 days post-vaccination and the frequencies of antigen specific CD8^+^ T cells were measured using tetramers for NP_366-374_, an immunodominant NP-specific CD8^+^ T cell epitope *via* flow cytometry (**Supplementary Figures 3A, B**). The frequencies of NP_366-374_ tetramer binding CD8^+^ T cells were not statistically different between WT, *cGAS^-/-^* and *STING^-/-^* mice (**Figure 2A**) indicating that *cGAS* and *STING* are not required for DNA vaccine induction of antigen-specific CD8^+^ T cells. To determine if *STING* impacts effector functions of CD8^+^ T cells, we next measured the frequencies of polyfunctional CD8^+^ T cells expressing one or more cytokines IL-2, TNF-α, IFN-γ and the cytolytic markers CD107a/Granzyme B following stimulation with NP_366-374_ by intracellular cytokine staining (ICS) then flow cytometry (**Supplementary Figure 4**). To compare CD8^+^ T cell effector functions, a polyfunctionality index score was calculated for each mouse. The polyfunctional index enumerates cellular polyfunctionality as a one-dimensional value where greater value is given to CD8^+^ T cells that express more functions (17). CD8^+^ T cells in *STING^-/-^* mice exhibited significantly lower polyfunctional index scores when compared to WT and *cGAS^-/-^* mice (**Figures 2A, B**). These data suggest that *STING* and *cGAS* are not required for the generation of antigen-specific CD8^+^ T cells, but *STING* is required for the induction of polyfunctional CD8^+^ T cell responses.

### *STING* Expression Within Conventional Dendritic Cells Is Not Required for of DNA Vaccine Induction of CD8^+^ T Cell Responses

**Figure 3:**
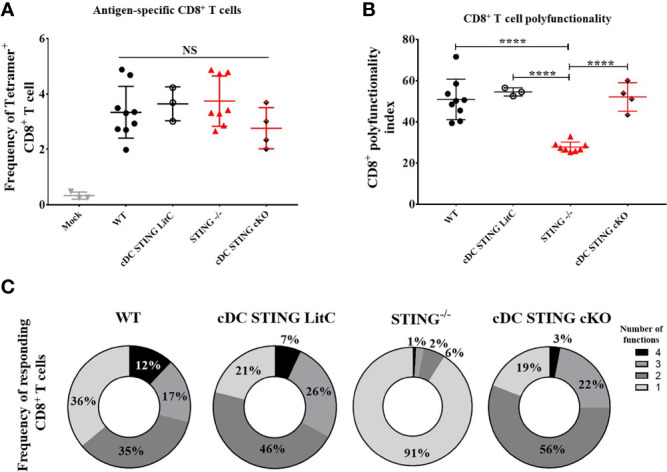
*STING* is not required within conventional dendritic cells to mediate DNA vaccine induction of CD8^+^ T cell responses. WT, *STING^-/-^, zbtb46^Cre^ xSTING^fl/fl^* (*cDC STING cKO)* and *cDC STING littermate control* (*cDC STING LitC*) mice were IM/EP vaccinated with pNP. Mice were sacrificed 21-days post-vacccination and splenocytes collected. **(A)** Tetramer positive cells within CD8^+^ T cells were measured for each genotype using a NP immunodominant peptide (NP_366-374_) containing tetramer as described above and shown in **Supplementary Figure 3C**. **(B)** Polyfunctional scores were determined by stimulating splenocytes overnight with 1μg/ml of NP_366-374_, staining for IL-2, TNF-α, IFN-γ and CD107a/GranzymeB and analysis via flow cytometry and Boolean gating as shown in **Supplementary Figure 4**. Shown are CD8^+^ T cells’ polyfunctionality index scores. **(C)** Pie charts show the relative average proportion of responding CD8^+^ T cells producing at least one immune function (IL-2, TNF-α, IFN-γ and/or CD107a/GranzymeB) after NP_366-374_ stimulation. Three independent experiments were performed consisting of 3-9 mice; representative data are the average ±SSD 3-9 mice/genotype. Groups were compared using a one-way ANOVA, ****p < 0.0001, and NS, not significant.

Conventional dendritic cells (cDCs) are important for the generation of DNA vaccine induced Ab and CD8^+^ T cell responses (10–14), therefore, we hypothesized that *STING* within *cDCs* may be required to mediate DNA vaccine induction of Th1 and CD8^+^ T cell responses. To test this, we generated mice that lacked *STING* in cDCs (*cDC STING cKO*), but not in plasmacytoid DCs (19). WT, *STING^-/-^*, *cDC STING cKO* and *cDC STING littermate control* (*cDC STING LitC*) mice (to control for the potential deleterious effects on *STING* due to the introduction of flox sites) were vaccinated by IM/EP delivery of 10μg of pNP. Mice were sacrificed 21 days post-vaccination and splenocytes were isolated to measure Ag-specific CD8^+^ T cell responses by NP_366-374_ tetramer staining (**Supplementary Figures 3A, C**) and polyfunctional CD8^+^ T cell responses by ICS and flow cytometry, as described above. WT, *STING^-/-^*, *cDC STING cKO* and *cDC STING LitC* mice had similar frequencies of tetramer binding CD8^+^ T cells (**Figure 3A**). In addition, WT, *cDC STING cKO* and *cDC STING LitC* mice developed similar levels of polyfunctional CD8^+^ T cell responses (**Figures 3B, C**). Together, these results indicate that while *STING* is required for induction of polyfunctional CD8^+^ T cell responses (**Figure 2**), it is not required within cDCs (**Figure 3**).

### DNA Vaccinated *cDC STING cKO* Mice Express Normal Levels of Pro-Inflammatory Cytokines

**Figure 4:**
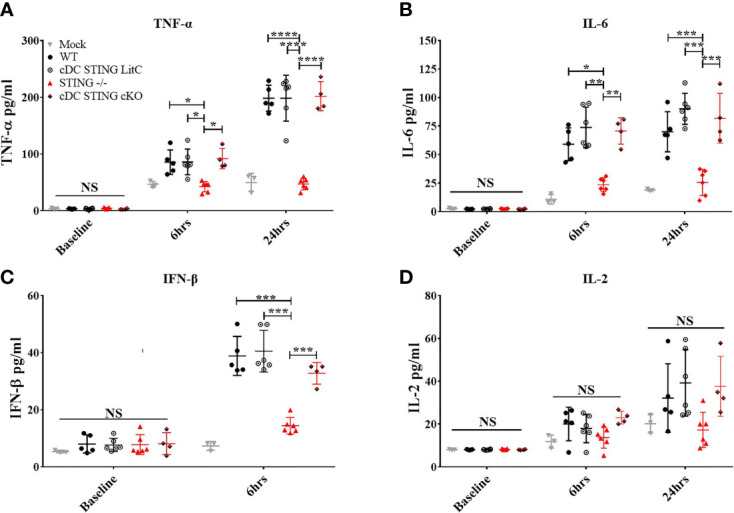
*STING* is required for DNA vaccine induction of innate pro-inflammatory cytokines, but not within cDCs. WT, *STING^-/-^, cDC STING cKO*, and *cDC STING LitC* mice IM/EP vaccinated with pNP. Sera was collected prior to vaccination (baseline), 6 hours (6hrs) and/or 24 hours (24hrs) post-vaccinations, and sera concentrations of **(A)** TNF-α, **(B)** IL-6, **(C)** IFN-β and**(D)** IL-2 were calculated. Three independent experiments were carried out consisting of 3-6 mice; representative data are the averages± SD of 3-6 mice/genotype. At each timepoint a one-way ANOVA was performed, *p < 0.05, **p < 0.01, ***p < 0.001,****p < 0.0001 and NS, not significant.

The production of pro-inflammatory cytokines influences the generation of Th1 or Th2 responses and CD4^+^ and CD8^+^ T cell effector functions (20–23). To determine if *STING* within cDCs influenced the induction of pro-inflammatory cytokines, WT, *STING^-/-^*, *cDC STING cKO* and *cDC STING LitC* mice were vaccinated with pNP *via* IM/EP delivery. Serum concentrations of pro-inflammatory cytokines were measured prior to vaccination, at 6 hours and at 24 hours post-vaccination. A control group of mice were mock treated by IM injection of saline followed by IM/EP to control for the induction of pro-inflammatory cytokines induced by the vaccination method. Consistent with previous studies (9), we found that sera from *STING^-/-^* mice has significantly lower levels of TNF-α (**Figure 4A**), IL-6 (**Figure 4B**) and IFN-β (**Figure 4D**) when compared to sera from WT, *cDC STING cKO* and *cDC STING LitC* mice. *STING^-/-^* mice also had lower levels of IL-2 (**Figure 4D**) compared to sera from WT, *cDC STING cKO* and *cDC STING LitC* mice, but these differences did not reach statistical significance. These data suggest that *STING* mediates the induction of pro-inflammatory cytokines after DNA vaccination, but not within cDCs.

### *STING* Is Required Within cDCs for DNA Vaccine Induction of Th1-Associated IgG_2C_ Antibody Responses

**Figure 5:**
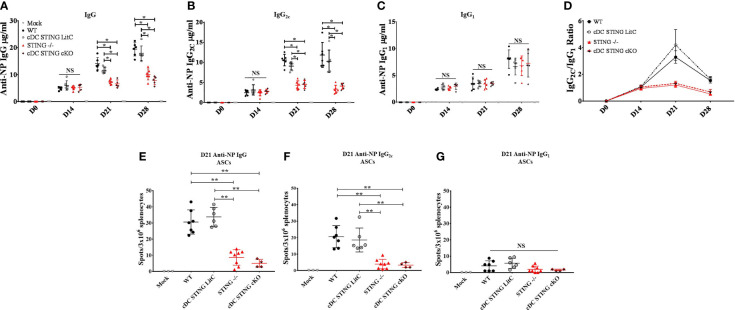
*STING* is required within conventional dendritic cells for DNA vaccine induction of antigen-specific IgG_2C_ antibody responses. WT, *STING^-/-^, cDC STING cKO* and *cDC STING LitC* mice IM/EP vaccinated with pNP. Sera were collected prior to vaccination (D0), 14 days (D14), 21 (D21) and 28 days post-vaccination (D28). Anti-NP **(A)** IgG, **(B)** IgG_2C_ and **(C)** IgG_1_ antibody responses was measured by ELISA as described above. **(D)** The Th1:Th2 ratio was calculated as the ratio of IgG_2C_:IgG_1_. A Th1:Th2 score >1 indicates a predominantly Th1 responses with a higher score indicating a strong Th1 response. Antibody secreting cells (ASCs) in WT, *STING^-/-^, cDC STING cKO* and *cDC STING LitC* mice vaccinated with pNP were measure 21 days post-vaccination by B cell ELIspot. Shown are **(E)** IgG, **(F)** IgG_2C_ and **(G)** IgG_1_ ASCs. Three independent experiments were performed consisting of 3-7 mice; representative data shown are the average± SD of 3-7 mice/genotype. A one-way ANOVA was employed to compare groups, *p < 0.05, **p < 0.01 and NS, not significant.

Since our data indicated that *STING* is required to mediate optimal IgG response and specifically, Th1-associated IgG_2C_ Abs, we next investigated if *STING* within cDCs was necessary to generate these responses. WT, *STING^-/-^*, *cDC STING cKO* and *cDC STING LitC* mice were vaccinated with pNP *via* IM/EP delivery. IgG, IgG_1_ and IgG_2C_ Ab responses were measured by ELISA prior to vaccination (D0), and at 14-, 21- and 28- days post-vaccination. *STING^-/-^* and *cDC STING cKO* mice developed significantly lower NP-specific IgG (**Figure 5A**) and IgG_2C_ (**Figure 5B**) 21- and 28-days post-vaccination, but comparable IgG_1_ (**Figure 5C**) responses when compared to WT and *cDC STING LitC* mice. Consequently, *STING^-/-^* and *cDC STING cKO* mice also had lower Th1/Th2 ratios (IgG_2C_/IgG_1_) than the control mice (**Figure 5D**) indicating a lower Th1 response. Together, these results show that *STING* within cDCs is required to induce Th1 (IgG_2C_) type Ab responses. To determine if the lower IgG_2C_ Ab responses observed in *STING^-/-^* and *cDC STING cKO* mice was due to a reduction in the number of Ab-secreting B cells (ASCs), the number of NP-specific IgG (**Figure 5E**), IgG_2C_ (**Figure 5F**) and IgG_1_ (**Figure 5G**) ASCs were quantified 21 days post-vaccination *via* a B cell ELIspot assay. *STING^-/-^* and *cDC STING cKO* mice developed comparable number of IgG_1_ ASCs, but significantly fewer IgG and IgG_2C_ ASCs than the control mice, further supporting our findings that *STING* is required within cDCs to induce IgG_2C_ secreting B cells following DNA vaccination.

### *STING* Is Required for DNA Vaccine Induction of Antigen-Specific Th1 CD4^+^ T Cell Responses

**Figure 6:**
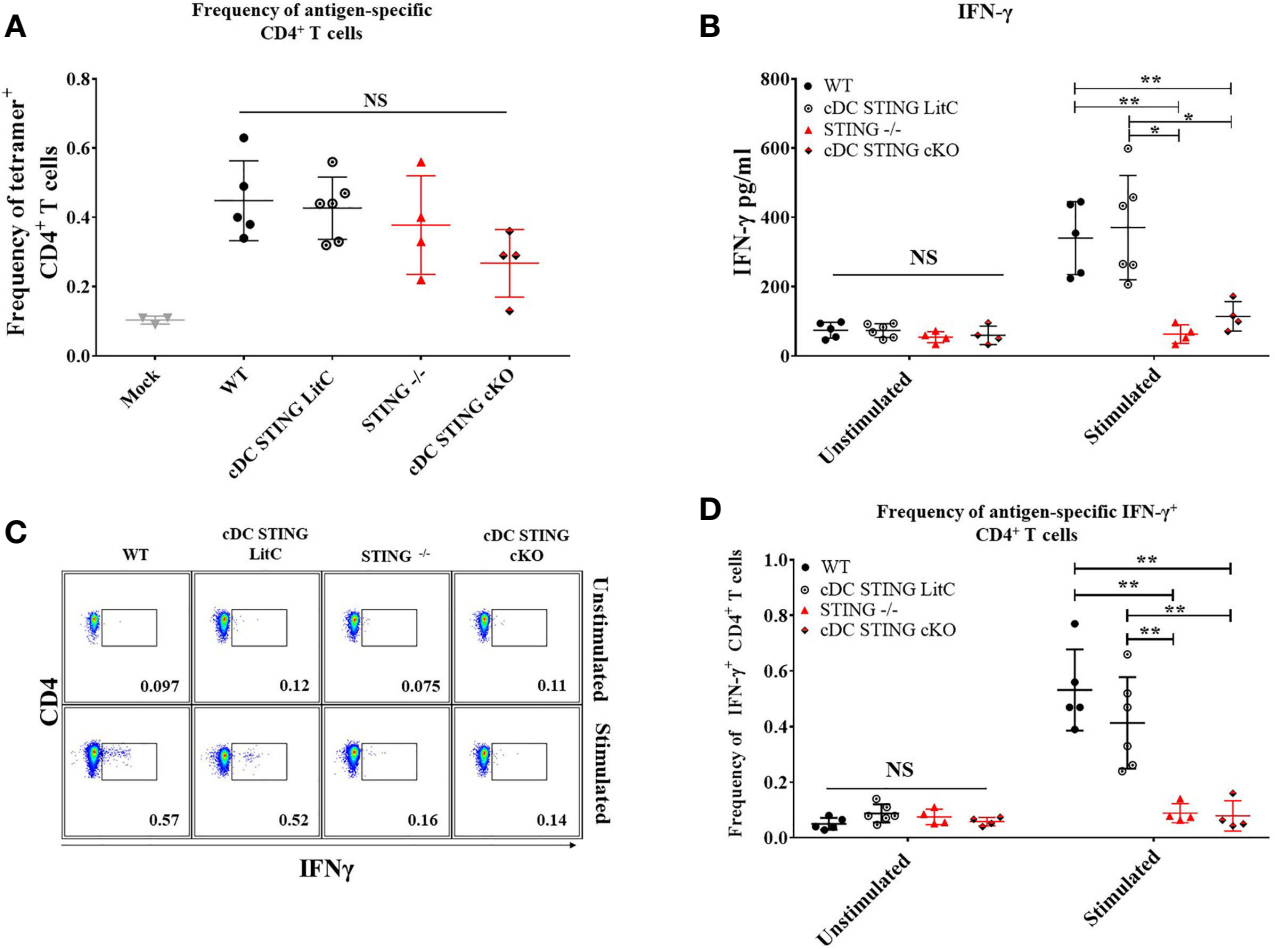
DNA vaccines require *STING* within cDCs to induce vaccine generated IFN-γ^+^CD4^+^ T cells and IFN-γ production. WT, *STING^-/-^*, *cDC STING cKO* and *cDC STING LitC* mice were IM/EP with pNP. Mice were sacrificed 21 days post-vaccination and splenocytes were isolated. **(A)** The frequency of tetramer positive cells within CD4^+^ T cells were measured for each genotype using a MHC-II tetramer that presents the NP immunodominant peptide (NP_311-325_) for C57bl/6. Addtionally, splenocytes were stimulated with NP_311-325_ to evaluate the cytokines produced by splenocytes. **(B)** IFN-γ production by CD4^+^ T cells was measured by stimulating splenocytes with NP_311-325_ peptide in vitro and analyzing the supernatants. Production of IL-2, IL-4, IL-6, IL-10, and TNF-α were also measured (**Supplementary Figure 6**). **(C, D)** frequencies of IFN-γ^+^CD4^+^ T cells measured by flow cytometry. **(C)** Gating scheme for detection of IFN-γ^+^CD4^+^ T cells by flow cytometry. **(D)** Frequencies of IFN-γ^+^CD4^+^ T cells. Three independent experiments were performed consisting of 3-6 mice; representative data shown are the average± SD of 3-6 mice/genotype. A one-way ANOVA was employed to compare groups, *p < 0.05, **p < 0.01 and NS, not significant.

To determine the role of *STING* within cDCs for DNA vaccine induction of CD4^+^ T cell responses WT, *STING^-/-^*, *cDC STING cKO* and *cDC STING LitC* mice were IM/EP-vaccinated with pNP and the frequencies of NP-specific CD4^+^ T cells binding the NP-specific CD4^+^ T cell epitope, NP_311-325_, were measured by tetramer staining and flow cytometry (**Supplementary Figure 5**) 21 days post-vaccination. Both *STING^-/-^* and *cDC STING cKO* mice exhibited a trend toward lower frequencies of Ag-specific CD4^+^ T cells, but these differences were not significantly different from control groups (**Figure 6A**). To determine the impact of *STING* within cDCs on the induction of Th1 CD4^+^ T cell responses, we evaluated cytokines produced by splenocytes stimulated with a C57Bl/6 CD4^+^ T cell NP immunodominant peptide NP_311-325._ Splenocytes were isolated 21 days post-vaccination, stimulated overnight with 1μg/ml NP_311-325_ and then concentrations of Th1 (IFN-γ, TNF-α, IL-2 and IL-6) and Th2 (IL-4) cytokines were analyzed in the supernatants using a flow-based cytokine detection kit (24). Overall, we observed no significant differences in the production of TNF-α, IL-2, IL-4 and IL-6 (**Supplementary Figure 6**). We also measured IL-10 [a marker of Tregs (25)] and IL-17a cytokines but observed no differences between the groups suggesting that *STING* may have limited impact on the induction of Tregs or Th17 T cells following DNA vaccination (**Supplementary Figures 6D, F**) but more studies are required to determine if *STING* impacts these and other T cell subsets. However, *STING^-/-^* and *cDC STING cKO* splenocytes produced significantly less IFN-γ when compared to WT and *cDC STING LitC* mice (**Figure 6B**), suggesting a role for *STING* within cDCs in the induction of Th1/IFN-γ-producing CD4^+^ T cell responses. To further investigate this, CD4^+^ T cells from *STING^-/-^* and *cDC STING cKO* mice were stimulated *in vitro* with the NP CD4^+^ T cell epitope, NP_311-325_, stained for IFN-γ by ICS and then analyzed by flow cytometry (**Figure 6C**). *STING^-/-^* and *cDC STING cKO* mice developed significantly lower frequencies of IFN-γ^+^CD4^+^ T cells when compared to WT and *cDC STING LitC* mice (**Figure 6D**), further indicating that *STING* is required within cDCs for DNA vaccine induction of Th1 CD4^+^ T cell responses.

## Discussion

A hallmark of DNA vaccine in their ability to induce a strong Th1-biased immune response (1–5). However, the mechanisms that influence this Th1 bias are unclear. Here, we investigated the role of *cGAS* and *STING* in DNA vaccine induction of Th1 and Th2-associated Ab and T cell responses. Consistent with previous findings (9), we found that *STING*, but not *cGAS*, is required for DNA vaccine induction of IgG Ab responses. Strikingly, we further observed that *STING*, but not *cGAS*, is required for DNA vaccine induction of Ag-specific IgG_2C_ (Th1) Ab responses, but neither *STING n*or *cGAS* is required to induce Ag-specific IgG_1_ (Th2) Ab responses (**Figure 1**). Additionally, we found that *STING* is required for DNA vaccine induced IgG_2C_ responses independent of the modality of vaccine delivery since gene gun (GG) vaccinated *STING^-/-^* mice had significantly lower IgG_2C_ concentrations compared to control mice, but generated normal concentrations of IgG_1_. The lack of significant impact of *STING* on the total IgG responses following GG delivery of DNA vaccines is likely due to GG inducing predominantly IgG_1_ responses whereas IM induced primarily IgG_2_ antibody responses (26), a finding consistent with our data showing that total IgG in GG vaccinated mice primarily consist of IgG_1_. These results provide the first evidence that *STING* is required for DNA vaccine induction of Th1 associated IgG_2C_ Ab, but not Th2 associated IgG_1_ Ab responses.

Th1 responses play a role in programming CD8^+^ T cell memory and effector functions (27) yet we found that neither *STING* nor *cGAS* were required for DNA vaccine induction of Ag-specific CD8^+^ T cell responses. Instead, our results show that *STING*, but not *cGAS*, is required for DNA vaccine induced polyfunctional CD8^+^ T cell responses (**Figure 2**). Our findings therefore provide new insight into the role of *STING* in mediating the ability of DNA vaccines induction of polyfunctional CD8^+^ T cell responses and suggest a strong link to the induction of Th1 responses, but additional studies are still needed to identify the precise mechanism underlying the ability of DNA vaccines to induce robust CD8^+^ T cell responses.

Previous studies highlight a critical role of DCs in coordinating both Ab and CD8^+^ T cell responses induced by DNA vaccines (10–14). Using mice with *STING* selectively absent in cDCs (*cDC STING cKO*), our studies surprisingly showed that *STING* is not required within cDCs for DNA vaccine induction of polyfunctional CD8^+^ T cell responses. The discrepancy between our findings that show *STING* is required for the induction of polyfunctional CD8^+^ T cell responses, but not within cDCs suggest that *STING* within other cell types likely mediates the ability of DNA vaccines to induce polyfunctional CD8^+^ T cells. In support of this, we found that the production of pro-inflammatory cytokines (IL-6, TNF-α, and IFN-β) after DNA vaccination was significantly diminished in *STING^-/-^* mice but similar in control mice and mice lacking *STING* in cDCs (**Figure 4**). Plasmacytoid dendritic cells (pDCs), a DC subset that still possess *STING* in *cDC STING cKO* mice, may be a potential DC subset that is producing IFN-α/β post-DNA vaccination, which subsequently aids in CD8^+^ T cell effector programming (15, 28). Additionally, studies suggest that DNA vaccine transfected myocytes produced some IFN-β albeit in significantly lower quantities (29) and could contribute to induction of polyfunctional CD8^+^ T cell responses. Interestingly we found that *STING^-/-^* mice developed similar frequencies of Ag-specific CD8^+^ T cells even though *STING^-/-^* mice exhibited somewhat lower concentration of IL-2 following DNA vaccination. The reason for this possible discrepancy is not clear but may be due to differences in the threshold of IL-2 production that are required to generate Ag-specific CD8^+^ T cells responses vs. facilitate their polyfunctionality. Unlike CD4^+^ T cells, activated CD8^+^ T cells can undergo maximum proliferation when exposed to even low concentrations of IL-2 (30, 31). Thus, it is possible that the somewhat lower amounts of IL-2 production in *STING^-/-^* mice is still above the threshold required for expansion of CD8^+^ T cells, but is not sufficient to program polyfunctional CD8^+^ T cells which relies on type I IFN and other factors more significantly impacted by *STING*.

In contrast, we found that *STING* is required within cDCs for DNA vaccine induction of Th1 associated IgG_2C_ Ab responses (**Figure 5**), a result that suggests that *STING* within cDCs may play a key role in B cell IgG class switching. Indeed, we found that DNA vaccinated mice lacking *STING* within cDCs developed lower frequencies of IFN-γ producing CD4^+^ T cells that are known to play a key role in promoting B cell IgG class switching to IgG_2C_ production (**Figure 6**) (32–37). These results are consistent with previous studies showing CD4^+^ T cells in *STING^-/-^* mice express significantly lower IFN-γ than WT mice (32), but further extends these results to show, for the first time, that *STING* is specifically required within cDCs for DNA vaccine induction of Th1 IFN-γ producing CD4^+^ T cells. Additional studies are needed to elucidate if the absence of *STING* in cDCs results in atypical cDC and CD4^+^ T cell interactions. For example, a reduction in Th1 polarizing cytokines (i.e. IL-12 and/or IL-23) produced by cDCs could adversely impact Th1 T cell polarization (38–43) . Since *STING* is absent in both CD8^+^ and CD8^-^ cDCs in *cDC STING cKO* mice, additional studies will be needed to determine if *STING* is required in both of these cDC subsets to mediate DNA vaccine induced Th1 immune responses.

Taken together, these data elucidate a clearer role for *STING* in mediating DNA vaccine induction of Ab and CD8^+^ T cell responses. Our results show that *STING* is not required for the induction of Ag-specific CD8^+^ T cell responses but is required to induce polyfunctional CD8^+^ T cells responses. Although our studies show that *STING* is dispensable within cDCs for DNA vaccine induction of polyfunctional CD8^+^ T cells, *STING* is required within cDCs for the generation of Th1 (IgG_2C_) Ab responses and Th1 IFN-γ producing CD4^+^ T cells. Taken together, these results provide new insight into the mechanisms whereby DNA vaccines induce Th1 responses. We propose that upon DNA vaccination, *STING* within cells other than cDCs triggers the production of pro-inflammatory cytokines (IL-6, TNF-α and IFN-β) that are critical for the induction of polyfunctional CD8^+^ T cell responses. Alternatively, *STING* may facilitate apoptosis in DNA transfected cell and cross-presentation to CD8^+^ T cells by cDCs. Indeed, cross-presentation has been shown to be a mechanism whereby intramuscular delivered DNA vaccines induce CD8^+^ T cell responses (44). Furthermore, recent studies have shown that *STING* activation can induce apoptosis (45).

The ability of DNA vaccines to induce Th1-polarized Ab responses, Ag-specific T cell responses and polyfunctional CD8+ T cells responses is believed to be a key feature or the development of vaccines effective for a wide range of infectious diseases and cancers (33). Our findings reported here provide new insight into the underlying innate pathways required to mediate DNA vaccine immunogenicity and specifically, the role of *STING* in the induction of Th1 responses. Together, the results have implications for the development of new DNA vaccine strategies and genetic adjuvants to enhance or modulate DNA vaccine induction of Th1 immune responses. While our studies and previous studies confirm a requirement for *STING* to mediate DNA vaccine immunogenicity, it remains unclear if RNA based vaccines require *STING*, studies have shown that *STING^-/-^* mice are susceptible to RNA virus infections (46), a result that suggests *STING* may also be required to mediate RNA vaccine induced immune responses. However, additional studies have shown that RNA viruses can induce mitochondrial damage resulting in mitochondrial DNA release that in turn activates *STING* (47, 48) suggesting that *STING* may be indirectly required to mediate RNA vaccine induced immune responses. Additional studies are still needed to determine the host factors influencing RNA vaccine immunogenicity.

## Data Availability Statement

The raw data supporting the conclusions of this article will be made available by the authors, without undue reservation.

## Ethics Statement

The animal study was reviewed and approved by Institutional Animal Care and Use Committee.

## Author Contributions

JU-L designed these studies, preformed the experiments, interpreted the data and wrote the manuscript. KD collected mouse samples including sera, genotyping, and tissue collection and processing. KR and MO helped to perform some experiments and helped with data analysis. EA and DF managed the study and critically revised the manuscript. All authors contributed to manuscript revisions and approve of the submitted version.

## Funding

Funding for this study was support by National Institutes of Health (NIH) grant R01AI442257 and Washington Research Fund “A diametric genetic adjuvant strategy to maximize immunogenicity of gene gun vaccines”.

## Conflict of Interest

The authors declare that the research was conducted in the absence of any commercial or financial relationships that could be construed as a potential conflict of interest.

## Publisher’s Note

All claims expressed in this article are solely those of the authors and do not necessarily represent those of their affiliated organizations, or those of the publisher, the editors and the reviewers. Any product that may be evaluated in this article, or claim that may be made by its manufacturer, is not guaranteed or endorsed by the publisher.

## Abbreviation

IFN, interferon; Ab, antibody; Ag, antigen; hrs, hours; embryonic stem cells, ES; littermate control, Litc; cKO, conditional knock-out; MyD88, myeloid differentiation primary response gene 88; dsDNA, double stranded DNA; cGAS, cyclic GMP-AMP synthase; STING, stimulator of interferon genes; TBK1, TANK-binding kinase 1; IRF7, interferon regulatory factor 7.
