# Supplementary material for: STING Is Required in Conventional Dendritic Cells for DNA Vaccine Induction of Type I T Helper Cell- Dependent Antibody Responses
Source: Front Immunol. 2022 Apr 22;13:861710. doi: 10.3389/fimmu.2022.861710 (PMC9072870; doi:10.3389/fimmu.2022.861710)
Supplement: Supplementary file 1 [file DataSheet_1.docx]

**Supplemental table 1**

| Primer | Forward | Reverse | Product size |
| --- | --- | --- | --- |
| **For tail snip genotype** | |  |  |
| STING fl/fl | GTG GCT TTG AGA GAA AAG TGA CCT AAC | GGA CAC TGA ATG TCT GTG TCA GTC CC | 385 (flox);321 (WT) |
| CRE | CTG GTT GAG GAT GAG GAT GA | GCG CTG GAG TTT CAA TAC C | 267 (WT); mutant (202) |
| **For Genomic validation** |  |  |  |
| exon 6 | GGGAGCCGAAGACTGTACAT | CGCTGTTGGAATAAACCCGA | 521 (WT) 472 (KO) |

**Supplemental Figure 1**

**Validation that *zbtb46^Cre^x STING^fl/fl^ (cDC STING cKO)* mice lack *STING* in cDCs. (A)** Generation of a conditional allele, via a sequence replacement strategy to knock-out the *STING* gene. The construct contains LoxP sites that flank exon 6, a 2kb 5’ homologous, and a Neomycin (Neo) cassette flanked by frt sites for selective deletions. The Neo element allowed for positive selection in ES cells. After homologous recombination of the conditional KO construct, the Neo was excised Flp-e administration. The *STING* gene had normal expression until Cre-mediated deletion of exon 6. This recombination created a frameshift mutation that resulted in a non-functioning *STING* gene. Gating strategies used to isolate individual immune cells to measure relative *STING* expression via a BD FACS ARIA III cell sorter. (**B**) Lymphocytes were identified by excluding doublets using forward and side light scatter, then B/T cells were isolated (CD19^+^CD3^+^), then non-B/T cells were further gated to isolate cDCs (CD11c^+^MHC-II^hi^). (**C**) qRT-PCR analysis of *STING* exon 6 to verify deletion under Cre control. Splenocytes from *zbtb46^Cre^*, *STING^fl/fl^*¸ and *cDC STING cKO* mice were isolated via flow as described above, and RNA was extracted, made into cDNA, then the relative expression of *STING* was measured.


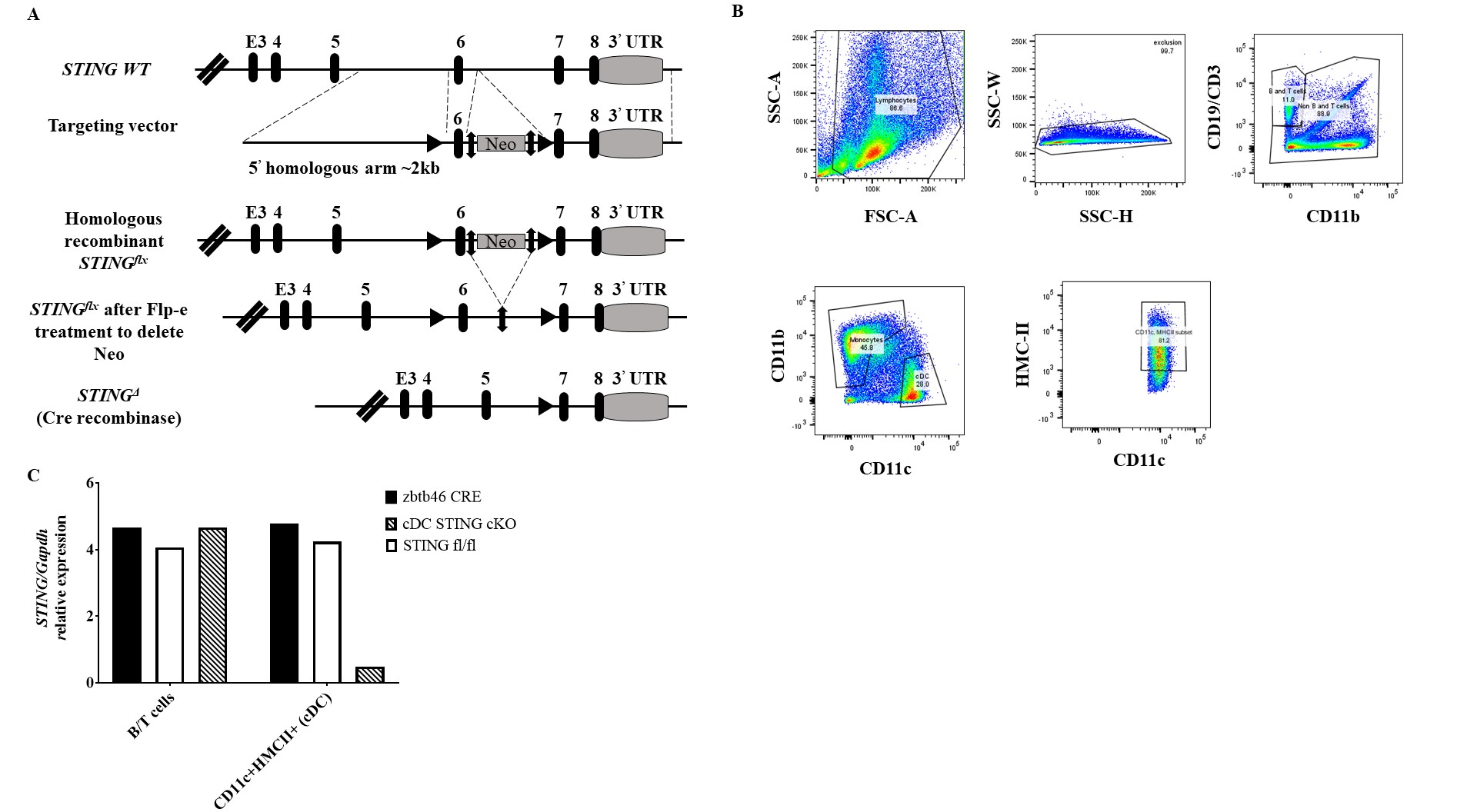


**Supplemental Figure 2**

***STING* is required for DNA vaccine induction of IgG_2C_ antibody responses independent of the vaccine delivery method.** Wild type (WT), *cGAS^-/-^* and *STING*^-/-^ mice were vaccinated with pNP using a gene gun (GG) to deliver vaccine DNA into the dermis of mice. Sera were collected prior to vaccination (D0), 14 days post-vaccination (D14), 21 days post-vaccination (D21) and 28 days post-vaccination (D28). (**A**) The concentration of anti-NP IgG was measured, though the overall responses were lower than IM/EP vaccinated mice. Anti-NP (**B**) IgG_1_ and (**C**) IgG_2C_ titers were measured 28 days post-vaccination*.* Three independent experiments were performed consisting of 5-10 mice; representative data shown are the average± SD of 5-6 mice/genotype. A one-way ANOVA was employed to compare groups, *p<0.05, **p<0.01 and ns, not significant.


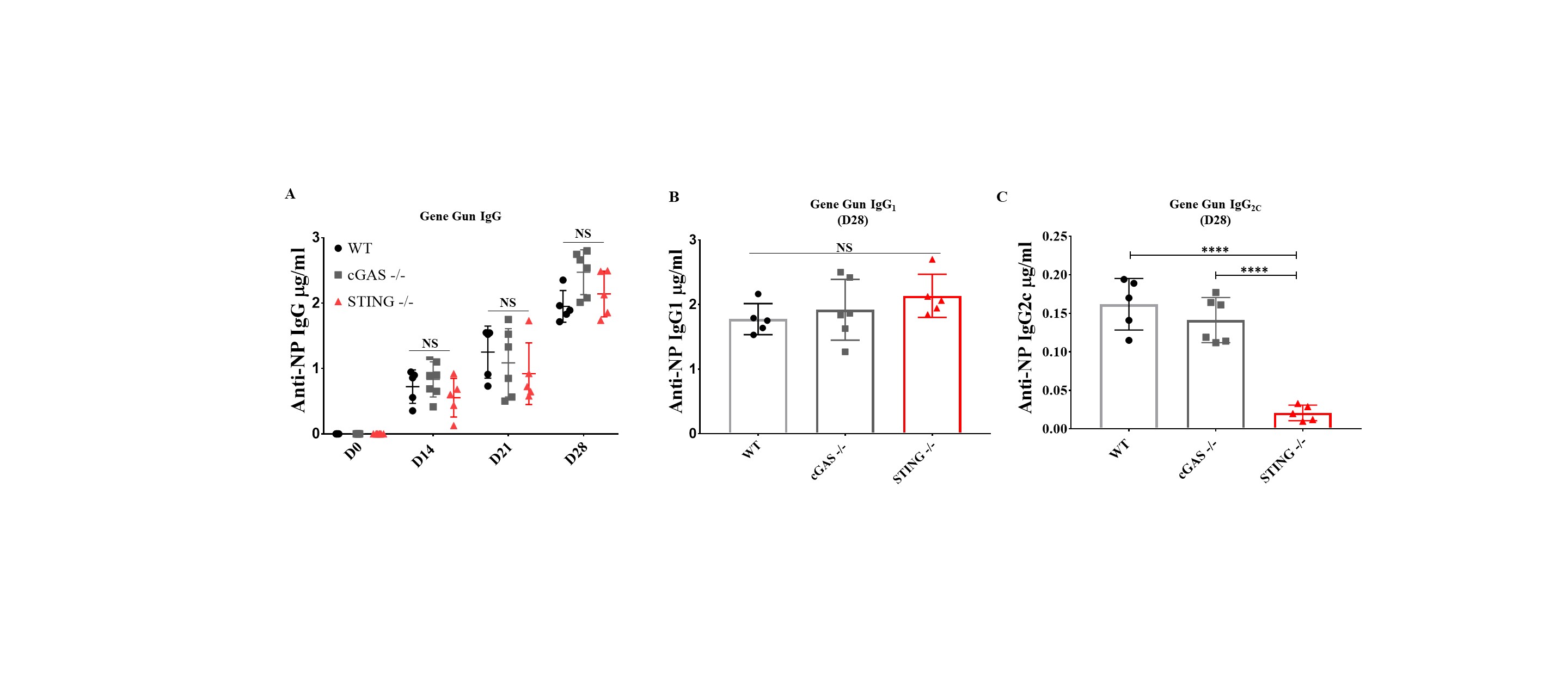

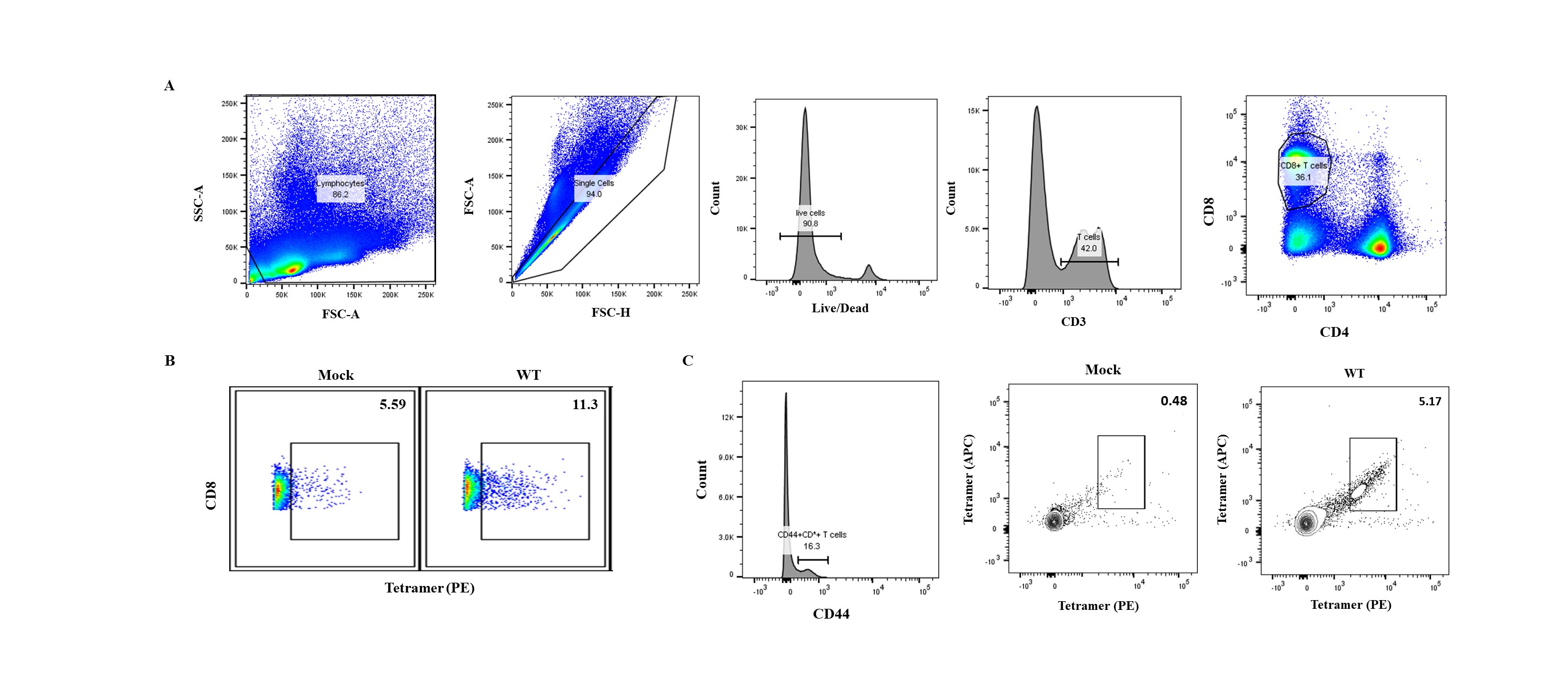


**Supplemental Figure 3**

Gating strategies used for evaluation of splenic tetramer binding CD8^+^ T cells. Representative gating strategy for splenocytes to identify tetramer binding CD8^+^ T cells. **(A)** Lymphocytes were identified by excluding doublets using forward and side light scatter, removing dead cells with a live/dead viability dye, then gating on CD3^+^ cells, followed by subsequent gating on T cell subsets CD4^+^ and CD8^+^ cells. **(B)** CD8^+^ Tetramer binding cells were identified using a PE-conjugated MHC-I tetramer (NP_366-374_) or (**C**) CD8^+^ T cells were further gated for CD44^+^ expression then Tetramer binding CD8^+^ T cells were identified using a PE- conjugated MHC-I tetramer (NP_366-374_) and a APC- conjugated MHC-I tetramer (NP_366-374_)


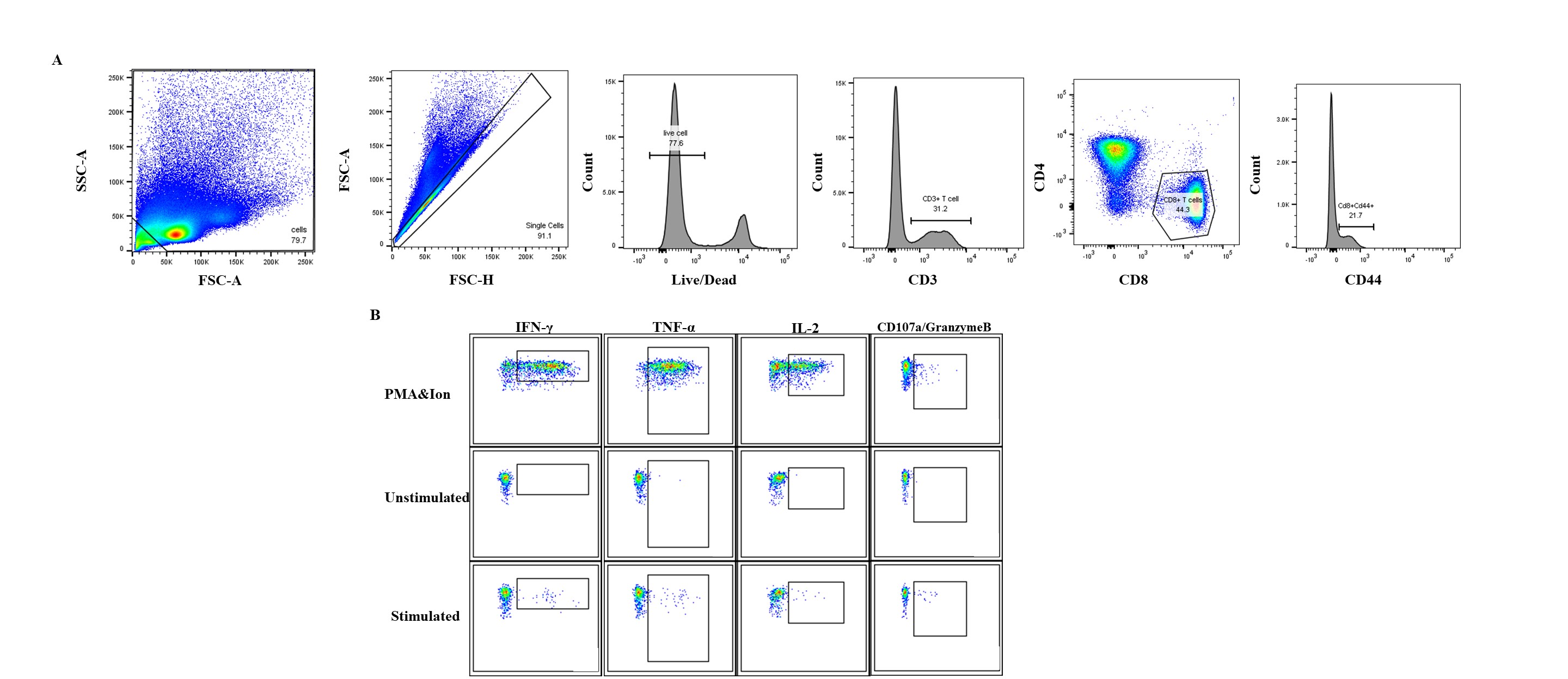


**Supplemental Figure 4**

Gating strategies used to evaluate CD8^+^ polyfunctionality in NP_366-374_ restimulated mice. **(A)** Lymphocytes were identified by excluding doublets using forward and side light scatter, removing dead cells with a live/dead viability dye, then gating on CD3^+^ cells, then T cell subsets CD4^+^ and CD8^+^ cells were identified, followed by subsequent gating for CD44^+^CD8^+^ T cells. **(B)** Representative gating of IFN-γ^+^, TNF-α^+^, IL-2^+^, and CD107aGranzymeB^+^ CD8 T cells. PMA and Ionomycin was used to set initial gates for each immune function outlined above, and once gates were set Boolean gating was used to evaluate polyfunctional CD8^+^ T cells.

**Supplemental Figure 5**

Gating strategies used for evaluation of splenic tetramer binding CD4^+^ T cells. Representative gating strategy for splenocytes to identify tetramer binding CD8^+^ T cells. **(A)** Lymphocytes were identified by excluding doublets using forward and side light scatter, removing dead cells with a live/dead viability dye, then gating on CD3^+^ cells, followed by subsequent gating on T cell subsets CD4^+^ and CD8^+^ cells, followed by gating for CD44^+^CD4^+^ T cells. **(B)** CD4^+^ Tetramer binding cells were identified using a PE-conjugated MHC-II tetramer (NP_311-325_).


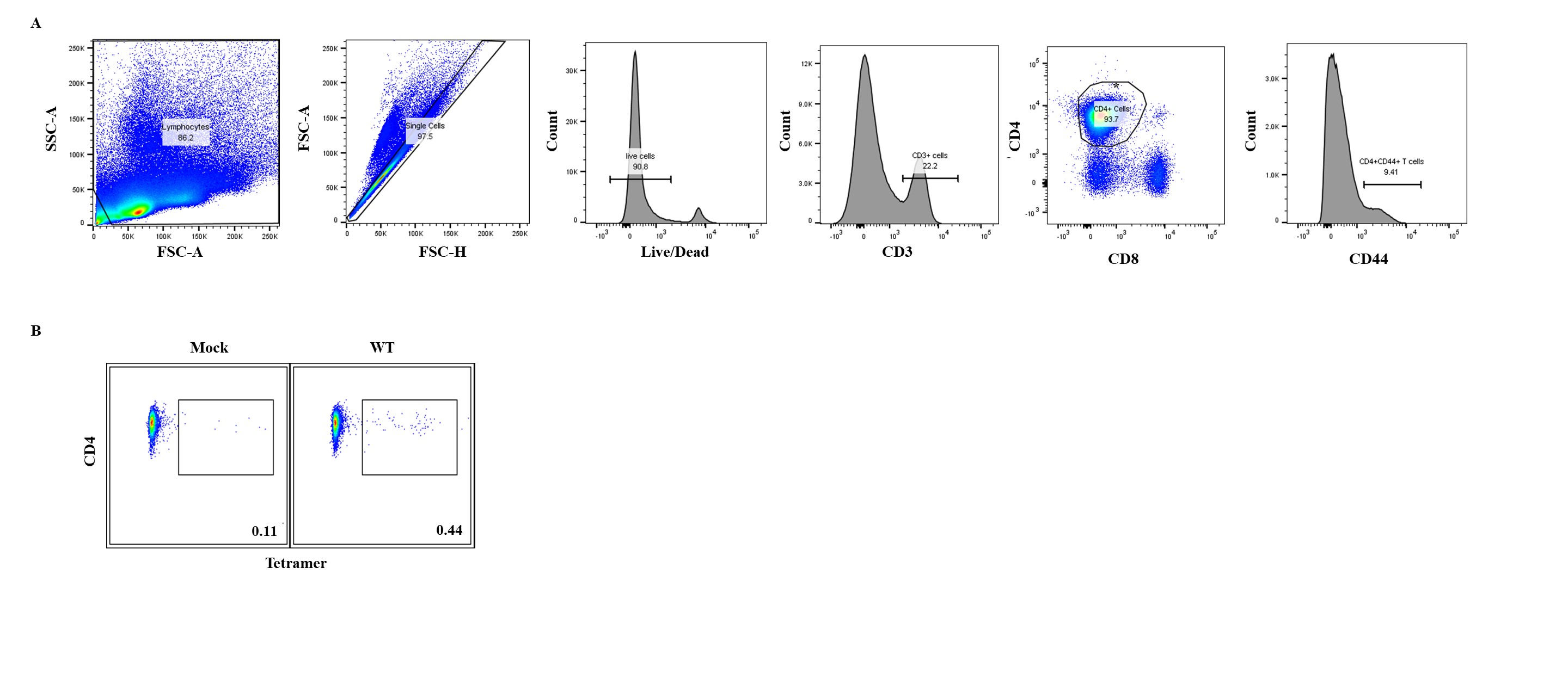

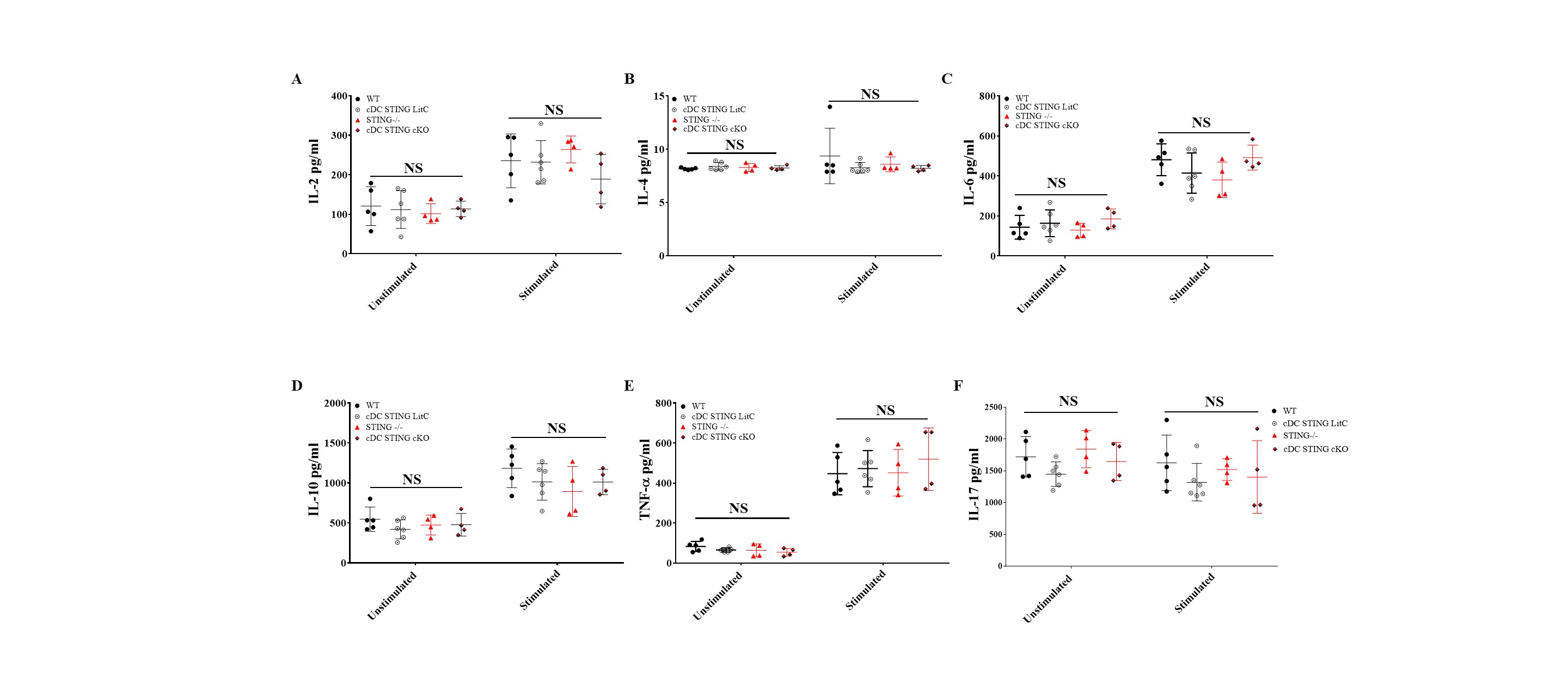


**Supplemental Figure 6**

WT, *STING^-/-^*, *cDC STING cKO* and *cDC STING LitC* mice were IM/EP with pNP, then mice were sacrificed 21 days post-vaccination and spleens were made into single cells suspensions. Cells were stimulated with NP_311-324_ peptide, and the concentration of (**A**) IL-2, (**B**) IL-4, (**C**) IL-6, (**D**) IL-10, (**E**) TNF-α and (**F**) IL-17a were measured from the supertantants. Frequencies of IFN-γ^+^CD4^+^ T cells. Three independent experiments were performed consisting of 3-6 mice; representative data shown are the average± SD of 4-6 mice/genotype. A one-way ANOVA was employed to compare groups for both stimulated and unstimulated conditions. ns, not significant
